# Psychometric properties of the earthquake knowledge questionnaire: Development for the Persian population

**DOI:** 10.1371/journal.pone.0331764

**Published:** 2025-10-09

**Authors:** Leila Jahangiry, Javad Babaei, Mitra Baghaeian, Hosna RashidiBirgani, Neda Gilani

**Affiliations:** 1 Tabriz Health Services Management Research Center, Tabriz University of Medical Sciences, Tabriz, Iran; 2 Department of Health Policy & Management, Road Traffic Injury Research Center, School of Management and Medical Informatics, Tabriz, Iran; 3 Student Research Committee, Tabriz University of Medical Sciences, Tabriz, Iran; 4 Department of Statistics and Epidemiology, Faculty of Health, Tabriz University of Medical Sciences, Tabriz, Iran; University of Vienna: Universitat Wien, AUSTRIA

## Abstract

**Background:**

Disaster management, as defined by the United Nations Office for Disaster Risk Reduction (UNDRR) involves foresighted planning to prevent, prepare for, respond to, and recover from disasters. Research proves that earthquake knowledge significantly contributes to preparedness behavior. The aim of this research is to develop a psychometrically valid questionnaire following UNDRR guidelines to assess earthquake awareness.

**Method:**

An exploratory sequential mixed-methods study was conducted between April and July 2024 in Tabriz, Iran. In the initial phase of the study, a comprehensive literature review and qualitative research were conducted to develop a preliminary item pool related to earthquake knowledge. Subsequently, the face validity, content validity, and construct validity of the items were assessed, followed by an evaluation of reliability through internal consistency, McDonald’s omega and test-retest methods. Exploratory Factor Analysis (EFA) using polychoric correlations and parallel analysis was conducted to determine factor structure. A polychoric correlation matrix was estimated from the sample of 350 respondents with 1000 iterations and using the principal factors method.

**Results:**

A polychoric correlation matrix was computed in R software (version 4.4.1) to estimate the non-linear relations between 14 ordinal items of the earthquake knowledge scale, of a sample of 350 participants. Parallel analysis using principal axis factoring determined three factors with adjusted eigenvalues greater than zero (observed eigenvalues: 7.5, 1.8, and 1.2 for the first, second, and third factor, respectively), which were retained as significant. The 14-item earthquake knowledge questionnaire (14-EKQ) was organized into three factors: Geological Knowledge, Mitigation Measures, and Preparedness Knowledge, reflecting various dimensions of earthquake awareness. EFA revealed that these three factors collectively accounted for 83.6% of the total variance. The RMSEA value of (RMSE = 0.070) falls within the acceptable range (≤ 0.08), indicating a reasonable fit. The CFI (CFI = 0.916) value is close to the threshold of 0.95, indicating a relatively good fit. The TLI value (TLI = 0.908) is slightly below the threshold of 0.95 but still suggests an acceptable fit. The internal consistency and internal correlation coefficient of EKQ indicated acceptable reliability.

**Conclusion:**

This study successfully developed and validated a 14-item EKQ. The scale was organized into three distinct factors: Geological knowledge, Mitigation measures, and preparedness knowledge, which collectively accounted for 83.6% of the total variance, demonstrating strong explanatory power. The use of polychoric correlation matrices, parallel analysis, and principal axis factoring (PAF) improved the factor extraction process by appropriately accounting for the ordinal nature of the questionnaire data. Model fit indices, including RMSEA and TLI, indicated an acceptable to good fit of the scale to the data. Additionally, the scale demonstrated acceptable reliability, as evidenced by internal consistency measures, McDonald’s omega, and test-retest reliability. The study’s EKQ makes a significant contribution to earthquake education by providing a validated tool to assess public awareness across geological knowledge, mitigation strategies, and preparedness knowledge, aligning with UNDRR guidelines. Further research is recommended to confirm its generalizability across diverse populations and contexts.

## Introduction

Natural disasters like earthquakes have enormous implications on public health and infrastructure across the world. Earthquakes, in particular, are noted for being unpredictable and having the ability to wreak havoc on large scales, affecting human life, property, and mental health [1,2]. Mortality caused by natural disasters has documented alarming figures, and developing nations have been disproportionately hit with a higher proportion. For example, in 2009, natural disaster mortality was four times greater than during the 1980s, and up to 40 times more deaths from natural disasters occur in developing countries than in developed countries [3]. Earthquakes have been a recurrent threat in Iran, with the historical record reflecting extensive human and economic losses [4–6]. Over the past century, more than 150,000 deaths have resulted from earthquakes, accounting for nearly half of the country’s disaster-related mortality [7]. Despite frequent and severe seismic activity, such as the Kermanshah (2017) and East Azerbaijan (2012) earthquakes, there remains a significant gap in the systematic assessment of earthquake-related public knowledge [8].

Disaster management, as defined by the United Nations Office for Disaster Risk Reduction (UNDRR), is the proactive planning to prevent, mitigate, prepare for, respond to, and recover from disasters, with public awareness being a key foundation for effective measures being public knowledge [9]. Public awareness forms the cornerstone of a successful preparedness strategy, as it enables individuals to include safer behavior before, during, and following earthquakes [10–12]. Studies in earthquake-prone nations like China [13], Japan [14], and Turkey [15] have all shown that higher earthquake knowledge is linked to more preparedness behavior, reducing vulnerability and enhancing resilience. A study [16] was conducted to determine the relationship between university students’ earthquake knowledge levels and their sustainable earthquake awareness. A moderate, significant positive correlation was observed between earthquake knowledge levels and sustainable earthquake awareness. Another study [17] aimed to assess Tehran citizens’ knowledge of safe emergency evacuation procedures during earthquakes, highlighting the need to improve safe emergency evacuation knowledge among Tehran residents. Further studies [16,18] were carried out to determine the relationship between earthquake knowledge levels and sustainable earthquake awareness, reinforcing the importance of these interconnected factors.

There are limited studies that have piloted the psychometric properties of earthquake knowledge questionnaires. The Earthquake Knowledge Assessment Scale (EKAS), which was created in Türkiye, offers a tested measure to determine knowledge related to earthquakes, factual and procedural knowledge that is needed for effective disaster preparedness [19]. Social-cognitive theories, however, as explained by Mulilis and Duval (1995) [20], emphasize that knowledge is not enough unless psychological factors such as perceived risk, self-efficacy, and behavioral intentions are engaged, all of which play a significant role in the act of preparedness. Moreover, Kohn et al.‘s (2012) systematic review of disaster preparedness scales illustrates methodological weaknesses in existing measures, showing the need for rigorous psychometric approaches, such as assessment of content validity, factor analysis, and reliability [21]. Together, these studies underscore the need for context-specific, psychometrically sound measures to evaluate earthquake knowledge in a holistic manner.

Measuring the level of knowledge about earthquakes and their facets is critical for effective disaster management, as put forth by the UNDRR. Despite the significant contribution of earthquake awareness, few studies have attempted to develop standardized questionnaires to assess it, indicating the need for well-designed population-specific and well-constructed measures. While several global tools exist for assessing disaster awareness, few have undergone rigorous psychometric validation, especially in the Persian context. Existing tools often lack cultural specificity or methodological rigor. Therefore, a contextually appropriate and psychometrically sound earthquake knowledge questionnaire is urgently needed to inform preparedness efforts in high-risk regions like Iran. This study aims to develop a psychometrically robust questionnaire to assess earthquake awareness and its dimensions, aligned to UNDRR guidelines for disaster management, to identify areas of public awareness deficits, and to guide targeted education campaigns to foster greater disaster preparedness, response, and recovery.

## Methods

### Design

This research was carried out between April and July 2024 in Tabriz, Iran, and uses the data analysis of a larger research study, utilizing the earthquake data to measure earthquake knowledge among a sample of Iranian participants.

### Participants and sampling

Participants were individuals aged more than 18 years from Tabriz, Iran, who were randomly sampled from the SIB (Integrated Health System) databases, which is a large Iranian electronic health data platform. The sample size was determined by considering the number of items (14) included in the study and multiplying the number of respondents by it, and the sample size was determined to be 350. For validation, we considered two sample sizes. The first sample (n₁ = 175) was subjected to exploratory factor analysis (EFA), and the second sample (n₂ = 175) was used for cross-validation of the confirmatory model obtained using the first sample. Therefore, 400 potential respondents were approached and requested to take part, and 350 respondents filled in the questionnaires. Table 1 outlines the features of the respondents in both samples.

**Table 1:** Demographic characteristics.

Variable	Total	Sample for EFA (n = 175)	Sample for CFA (n = 175)
Age (years)^*^	42.9 7(±14.27)	42.14 (±14.8)	43.45 (±13.7)
**Gender**			
Man	123 (35.6)	77 (44.3)	48 (28)
Woman	222 (63.4)	95 (54.6)	127 (72)
**Education level**			
Illiterate	12 (3.4)	6 (3.4)	6 (3.4)
Elementary	106 (30.3)	57 (32.9)	49 (28.5)
Secondary	100 (28.6)	47 (27.2)	53 (30.8)
University and higher	127 (36.2)	63 (36.4)	64 (36.6)
**Economic status**			
Good	10 (2.9)	6 (3.7)	4 (2.4)
Bad	237 (67.7)	114 (69.9)	123 (74.1)
Moderate	82 (24.9)	43 (26.4)	39 (22.3)
**Having earthquake experience**			
Yes	296 (88.6)	143 (85)	153 (92.2)
No	38 (11.4)	25 (15)	13 (7.8)

* Indices are Mean (±SD).

### Item development process

#### Systematic review.

To find existing earthquake knowledge questionnaires, a systematic review was carried out. PubMed, Scopus, Web of Science, and Persian databases (e.g., SID, Magiran) were searched with keywords like “earthquake knowledge,” “questionnaire,” “assessment tool,” and “psychometric properties.” English or Persian studies addressing earthquake knowledge assessment were included. Existing items and dimensions of the available tools were extracted and categorized to prepare the initial item pool (Initial items = 100).

#### Expert interviews.

To ensure compliance with UNDRR best practice principles in disaster management, eight disaster management, health education, and public health experts were individually interviewed using semi-structured interviews to evaluate the cultural appropriateness of the original item pool. The experts reviewed the items for relevance, comprehensibility, and completeness and gave feedback, suggested new items, or made proposals for revising the items so that the Earthquake Knowledge Questionnaire (EKQ) can effectively support disaster preparedness, response, and recovery efforts. This process improved the EKQ’s appropriateness for targeted education campaigns in seismically prone areas like Tabriz.

The initial draft of the EKQ was constructed by combining items from the systematic review and expert opinion. The questionnaire was constructed to be straightforward, readable, and culturally appropriate.

### Face and content validation

This research employs a mixed-methods design that incorporates both qualitative and quantitative viewpoints to enhance the evaluation process. Content validity was evaluated based on content validity index (CVI) and content validity ratio (CVR). The preliminary scale in the qualitative research was examined by a panel of 10 experts who were health educators, psychologists, and disaster management experts. They wanted to assess grammar, phrasing, and appropriateness of the scaling for every item [22]. In the quantitative phase, CVI and CVR were computed. The CVI assessed the simplicity, accuracy, and clarity of every item, and the experts scored them using a 4-point Likert scale: 1 = not relevant/clear/simple, 2 = somewhat relevant/clear/simple, 3 = relevant/clear/simple but needing some revisions, and 4 = very relevant/clear/simple [23]. Items with a CVI score below 0.80 were unacceptable and were excluded [24]. The CVR was employed to determine the importance of each item, and they were rated by experts as 1 = essential, 2 = useful but not essential, or 3 = not essential. Items with a score of 0.62 or more on the CVR were included and rated by a panel of 10 experts, according to Lawshe’s criteria [25].

Face validity was assessed through qualitative and quantitative techniques. The questionnaire was completed by a sample of 20 members of the general public, who were selected purposefully to reflect the multicultural backgrounds of earthquake-prone areas in Iran. The participants were requested to mark any ambiguous or unclear items. Some modifications were implemented to enhance clarity based on their feedback. During the quantitative phase, the impact score (frequency × importance) was utilized in determining the importance of each item.

Respondents also scored the importance of each item using a 5-point Likert scale. Items with an impact score of less than 1.5 that reported a mean frequency of less than 50% and a mean importance rating of less than 3 were considered inappropriate and were not included in further analysis [32]. This strenuous process assured the relevance, clarity, and appropriateness of the questionnaires to the target group.

The knowledge of earthquakes was measured using a scoring system that assigns points based on respondents’ answers to each item. Yes (Correct Answer): + 1 point; Awarded when the respondent provides a correct answer or demonstrates accurate knowledge. No (Incorrect Answer): −1 point; Deducted when the respondent provides an incorrect answer or demonstrates inaccurate knowledge. Don’t Know (Uncertain Response): 0 points; assigned when the respondent indicates uncertainty or lack of knowledge about the item.

### Statistical analysis

#### Construct validation.

Polychoric EFA was used to examine the data, due to the ordinal nature of the items in the questionnaire (3-point Likert scale: ‘Yes,’ ‘No,’ ‘Don’t know’). A polychoric correlation matrix was utilized rather than Pearson correlations because it considers the underlying continuous latent variables that are assumed to produce ordinal responses. It is a more appropriate method for categorical data with non-linear relationships that minimizes factor loading bias and increases accuracy for Likert-scale items [26,27].

Principal Axis Factoring (PAF) with oblique promax rotation was employed to extract factors, as opposed to Principal Component Analysis (PCA). PAF distinguishes between shared variance, as in the goal of discovering latent constructs, and does not incorporate item-specific variance. Oblique promax rotation was employed since the factors were apt to be related (e.g., understanding of fault lines would likely be connected to building safety), which would render more realistic and interpretable solutions than orthogonal rotations.

To identify the number of factors to extract, parallel analysis was performed with the “paran” package in R (version 4.4.1). A polychoric correlation matrix was estimated based on a sample of 350 respondents, 1,000 iterations, and the principal factors method. Factors with adjusted eigenvalues > 0 were retained. This was cross-validated against Kaiser’s rule (eigenvalues > 1), a scree plot, and Velicer’s Minimum Average Partial (MAP) test [28], all of which supported a three-factor structure and mitigated limitations of Kaiser’s rule [29]. Items were retained on the basis of their pattern of loadings and theoretical relevance, and items showing cross-loadings or loadings < |0.2| were deleted to maximize factor clarity.

The confirmatory Factor Analysis (CFA) model was developed based on the factor structure derived from the EFA. Given that the questionnaire items were ordinal variables with a three-point Likert scale, appropriate estimation methods were chosen to account for the non-normal nature of the data. Initially, a polychoric correlation matrix was calculated to more accurately represent the relationships between the ordinal indicators compared to Pearson correlation. Subsequently, CFA was conducted using Structural Equation Modeling (SEM) in Stata 17. Since the data were ordinal, the Diagonally Weighted Least Squares (DWLS) estimator was used, which is more suitable for categorical data than the Maximum Likelihood (ML) method. Missing data were handled using listwise deletion during the confirmatory factor analysis. Participants with incomplete responses to any of the 14 questionnaire items were excluded from the CFA to maintain the integrity of the DWLS estimation method.

Each item was assigned to the factor on which it loaded most strongly during the EFA. Maximum Likelihood Estimation (MLE) was employed to estimate the model parameters. The fit of the hypothesized model was assessed using several fit indices, including the Root Mean Square Error of Approximation (RMSEA), where values less than 0.08 indicate a reasonable fit and values below 0.05 indicate a close fit. The Comparative Fit Index (CFI) was also used, with values greater than 0.90 indicating an acceptable fit and values above 0.95 indicating a good fit. These indices were utilized to determine whether the model adequately represented the data.

#### Reliability assessment.

To assess reliability, we conducted an evaluation that included McDonald’s omega for each identified factor to determine internal consistency. A random sample of 20 participants was selected from the original study population to evaluate test-retest reliability. These participants completed the 14-item earthquake knowledge questionnaire during the first session (Time 1) and again after a two-week interval (Time 2). The intra-class correlation coefficient (ICC) was utilized to measure the stability of the scale, with ICC values of 0.40 or higher deemed acceptable [35].

The internal consistency of the earthquake risk perception questionnaire was evaluated through the analysis of Cronbach’s alpha coefficients for both the overall instrument and each factor. An alpha value of 0.70 or greater was considered acceptable. The statistical analysis for this study was carried out using Stata, version 17, and R software (version 4.4.1)

#### Ethical approval.

Informed written consent was obtained from all participants. All the methods included in this study are in accordance with the Declaration of Helsinki. The study received ethical approval from the Ethics Committee of Tabriz University of Medical Sciences (NO: IR.TBZMED.REC.1402.822).

## Results

In the current study, an initial pool of 100 items, identified through a literature review and qualitative research, was reviewed by the research team and experts. Following this review, 78 redundant and overlapping items were eliminated, leaving 22 items. These were subsequently organized into three thematic domains.

The research team evaluated and compared these 22 items to develop the initial version of the Earthquake Knowledge Questionnaire, consisting of 22 items. Subsequently, face and content validation were conducted to assess the questionnaire. During this process, eight items were removed because they did not meet the required CVI and CVR standards. As a result, 14 items in three factors were retained, which aligned with the established validity criteria and were deemed suitable for inclusion in the final version of the questionnaire.

Table 1 shows that the demographic characteristics of the EFA and CFA samples are generally comparable to the total sample, with some minor variations. For example, the CFA sample has a slightly higher proportion of women (72%) compared to the EFA sample (54.6%). Additionally, the CFA sample has a higher percentage of participants with earthquake experience (92.2%) compared to the EFA sample (85%).

Table 2 presents key statistical metrics for evaluating factors related to earthquake knowledge. The table was divided into three main factors. Factor 1: Geological knowledge (items 1–6): This factor focuses on respondents’ knowledge of the geological aspects of earthquakes, particularly the role of fault lines. The items in this domain assess understanding of the connection between fault lines and earthquakes (e.g., “Is there a connection between fault lines and earthquakes?” or “Is your residence located on or near a fault line?”). Factor 2: Mitigation measures (items 10, 11, 12, 13, and 14); This factor assesses knowledge related to structural safety and mitigation measures. The items in this domain evaluate understanding of the importance of building codes and retrofitting in reducing earthquake risks. Safe practices during an earthquake, such as securing furniture and avoiding hazardous areas, and the role of architectural design in minimizing earthquake damage. Factor 3: preparedness knowledge (items 7–9); this factor assesses knowledge related to safe locations in the workplace and home.

**Table 2:** Factor loading, percentage of variance, Cronbach’s alpha, and Omega McDonald of earthquake.

Factors	Items	Loading	Variance	Alpha Cronbach	McDonald’s omega	ICC
1	2. Is there a connection between fault lines and earthquakes?	0.958	58.52%	0.858	0.888	0.90
	1. Do you know what a fault line is?	0.934				
	3. Can it be said that earthquakes usually occur at or near fault lines?	0.931				
	4. Can it be said that the intensity and damage of earthquakes are greater at fault lines?	0.924				
	5. Do you know which areas of Tabriz are located on/near fault lines?	0.704				
	6. Is your residence located on or near a fault line?	0.284^*^				
2	12. Do you know what measures to take to increase the strength and safety of your building?	0.907	13.82	0.718	0.705	0.82
	11. Do you know where and from whom to obtain information about the safety and strength of your building?	0.897				
	10. Are you confident in the strength and safety of your building against earthquakes?	0.607				
	13. Do you know that heavy objects and items should be placed on the ground or close to the ground?	0.436				
	14. A gas stove or heater left on during an earthquake can cause an explosion.	0.431				
3	8. Are you aware of the safe locations in your workplace?	0.975	9.12	0.619	0.672	0.76
	7. Are you aware of the safe locations in your living area?	0.935				
	9. Do you usually follow news related to earthquakes?	0.282^*^				
	Total items = 14			0.836	0.826	0.827

* Item retained due to theoretical relevance despite lower factor loading, as discussed in the Limitations section.

The total variance explained by all items is 0.836, indicating that these factors significantly contribute to understanding earthquake awareness. The loading values indicate how strongly each item correlates with its respective factor. Higher values (closer to 1) suggest a stronger relationship. For example, the item “Is there a connection between fault lines and earthquakes?” has a loading of 0.958, indicating a strong correlation with Factor 1. The percentage of variance shows how much of the total variance in responses can be explained by each factor. Factor 1 (geological knowledge) explains 58.52% of the variance, which is the highest among the factors, emphasizing its importance in the overall model.

Cronbach’s Alpha and McDonald’s Omega are measures of internal consistency, indicating how well the items within each factor align with each other. Factor 1 has a Cronbach’s Alpha of 0.858 and McDonald’s Omega of 0.888, suggesting excellent reliability. Factor 2 shows a lower reliability (Alpha: 0.718, Omega: 0.705).

**Fig 1:**
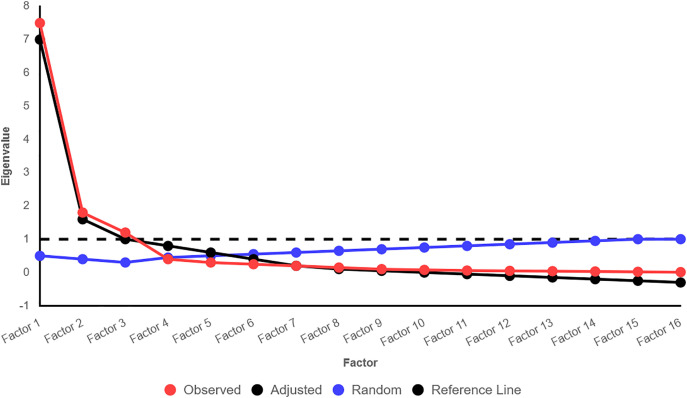
Scree plot.

**Fig 2:**
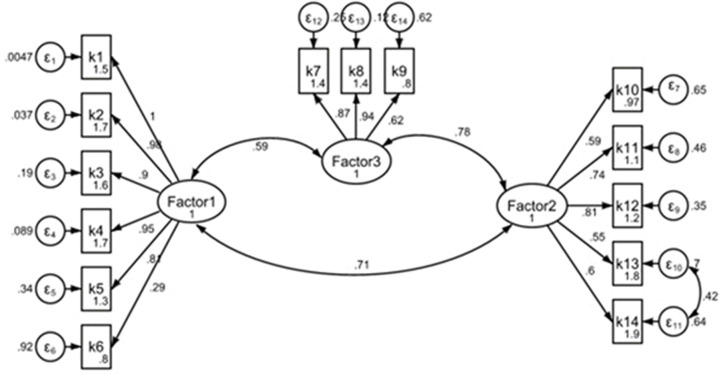
Confirmatory factor analysis.

The parallel analysis with the “paran” command revealed that three factors had more than zero adjusted eigenvalues and were thus selected as significant factors. The observed eigenvalues for the first three factors were 7.5, 1.8, and 1.2, respectively, whereas the adjusted eigenvalues were calculated to be 7.0, 1.6, and 1.0, respectively. After the fourth factor, the adjusted eigenvalues were less than zero. To confirm this selection, the Kaiser criterion was also examined and found that the observed eigenvalues of the first three factors were more than 1. The scree plot (Fig 1) also indicated an elbow at the third factor, according to the parallel analysis results. In this case, the lines of the found eigenvalues (red) for the first three factors were above the random eigenvalues (blue), while from the fourth factor onwards, these were close to or below the random line. Additionally, a horizontal reference line at 1 (black dashed line) was also included to compare with the Kaiser criterion (Fig 2).

The inter-item correlation matrix is (Table 3), representing the pair-wise correlations between 14 items (Knowledge1 to Knowledge14). The matrix reports strong correlations (0.927 to 0.965) among Knowledge1 to Knowledge 4, which suggests that these are very close to one another and measure the same thing regarding the construct (Geological knowledge). Knowledge 5 has weaker but still substantial correlations (0.698 to 0.709) with Knowledge 1 to Knowledge 4. On the other hand, Knowledge 6 is weakly correlated with all the other items (0.011 to 0.310) and is poorly related to the construct in general. Knowledge 7 and Knowledge 8 are strongly correlated with each other (0.877) but with others (e.g., 0.261 to 0.507) less so. Knowledge 9 to Knowledge 14 exhibit low correlations (e.g., 0.163 to 0.769) with themselves and earlier items.

**Table 3:** Inter-item correlation matrix.

	Knowledge1	Knowledge2	Knowledge3	Knowledge4	Knowledge5	Knowledge6	Knowledge7	Knowledge8	Knowledge9	Knowledge10	Knowledge11	Knowledge12	Knowledge13	Knowledge14
Knowledge1	1.000													
Knowledge2	0965	1												
Knowledge3	0.932	0.934	1											
Knowledge4	0.927	0.940	0.955	1										
Knowledge5	0.704	0.698	0.709	0.703	1									
Knowledge6	0.167	0.155	0.179	0.193	0.085	1								
Knowledge7	0.507	0.489	0.436	0.447	0.374	0.027	1							
Knowledge8	0.393	0.419	0.346	0.345	0.261	0.034	0.877	1						
Knowledge9	0.323	0.360	0.296	0.295	0.132	0.310	0.289	0.322	1					
Knowledge10	0.415	0.437	0.352	0.396	0.294	0.148	0.463	0.337	0.194	1				
Knowledge11	0.450	0.429	0.315	0.338	0.318	0.011	0.405	0.368	0.218	0.622	1			
Knowledge12	0.516	0.468	0.415	0.462	0.431	0.038	0.393	0.440	0.163	0.537	0.769	1		
Knowledge13	0.457	0.480	0.455	0.518	0.280	0.27	0.409	0.424	0.309	0.341	0.329	0.518	1	
Knowledge14	0.517	0.575	0.498	0.507	0.338	0.012	0.264	0.331	0.195	0.366	0.374	0.362	0.727	1

The Confirmatory Factor Analysis (CFA) was conducted to evaluate the fit of the hypothesized model to the observed data. The results are summarized below, along with an interpretation of the key fit statistics.

The significant chi-square value (p < 0.001) indicates a discrepancy between the hypothesized model and the saturated model. However, this statistic is sensitive to sample size and may not be the sole indicator of model fit. The significant chi-square value (Chi^2 ^= 182.521, p < 0.001) suggests that the hypothesized model fits better than the baseline model. The RMSEA value of (RMSE = 0.070) falls within the acceptable range (≤ 0.08), indicating a reasonable fit. The CFI (CFI = 0.916) value is close to the threshold of 0.95, indicating a relatively good fit. The TLI value (TLI = 0.908) is slightly below the threshold of 0.95 but still suggests an acceptable fit. The SRMR (SRMR = 0.076) value is below the threshold of 0.08, indicating a good fit.

## Discussion

The development and validation of the 14-item EKQ with its three-factor structure (Geological knowledge, Mitigation strategies, preparedness knowledge) demonstrate strong psychometric properties, making it a reliable and valid tool for assessing earthquake knowledge. The EKQ questionnaire has been derived from a comprehensive review and consultation with experts, by the UNDRR guidelines for managing disaster risk. These categories were selected to fill gaps in public knowledge through the incorporation of the key elements of earthquake knowledge, like tectonic fault understanding, structural safety provisions, and safe locations identification, all of which are critical for successful preparedness, response, and recovery operations.

The EKQ’s three-factor structure aligns well with the theoretical domains of earthquake knowledge, as identified in the literature. The factors, Geological knowledge, Mitigation measures, and preparedness knowledge, cumulatively explain 83.6% of the total variance, indicating that the questionnaire captures a significant portion of the variability in earthquake knowledge.

Factor 1 (Geological knowledge): This factor, comprising items 1–6, explains the highest proportion of variance (58.52%). The strong factor loadings (e.g., 0.958 for the item “Is there a connection between fault lines and earthquakes?”) suggest that this domain is a critical component of earthquake knowledge. This finding is consistent with the importance of understanding geological factors in earthquake knowledge. This aligns with studies emphasizing the role of understanding tectonic processes in earthquake preparedness [30].

Factor 2 (Mitigation measures): Items 10, 11, 12, 13, and 14 load onto this factor, reflecting knowledge about structural safety and mitigation measures [31]. The inclusion of items related to secondary hazards (e.g., fire) enhances the comprehensiveness of this factor [26], aligning with the multidimensional nature of earthquake preparedness. This factor captures both structural and non-structural aspects of safety, which are critical for effective preparedness [32].

Factor 3 (Preparedness knowledge): Items 7, 8, and 9, which address awareness of safe locations form this factor. While this factor has slightly lower reliability compared to Factor 1, it remains a key domain for assessing practical knowledge related to earthquake preparedness [27]. Although it includes fewer items, it contributes meaningfully to the overall construct of earthquake knowledge. The clear and interpretable factor structure supports the construct validity of the EKQ, indicating that it effectively measures the intended domains of earthquake knowledge.

The internal consistency of the EKQ was assessed using Cronbach’s Alpha and McDonald’s Omega, both of which indicate the reliability of the questionnaire. Factor 1 (Geological knowledge) with high reliability coefficients (Cronbach’s Alpha = 0.858, McDonald’s Omega = 0.818) suggests excellent internal consistency for this factor. This indicates that the items within this domain are highly correlated and measure the same underlying construct. This is consistent with the findings of Paton et al., who highlighted the importance of geological knowledge in disaster preparedness [33]. The reliability coefficients for Factor 2 (Mitigation measures) are acceptable but lower than those for Factor 1. This may reflect the diversity of items within this domain, which cover both structural and non-structural safety measures. The strong internal consistency of the factors suggests that the items are highly reliable in measuring geological knowledge about fault lines and their relationship to earthquakes.

Common questionnaires measuring earthquake knowledge are for general awareness or specific topics, e.g., preparedness or response [34,35], and not depth and specificity to capture a broad, actionable understanding. For instance, many measures assess basic knowledge, e.g., locating fault lines or the “Drop, Cover, and Hold On” process, but fail to capture important insights into structural vulnerability, secondary hazards, or long-term mitigation strategies [36]. The current study introduces a new approach by connecting profound knowledge (Geological knowledge) with practical, applicable applications (e.g., Mitigation measures). In contrast to previous tools, which rarely cover both sides [36].

The increasing frequency of high-impact earthquakes (e.g., Turkey-Syria 2023, Morocco 2023) underscores the urgent need for tools to assess actionable public knowledge. Existing measures often focus on general awareness rather than deep, preventable knowledge (e.g., Geological knowledge and Mitigation measures). Our study addresses this gap by providing a validated questionnaire tailored to regions with high seismic risk, where localized knowledge directly informs preparedness behaviors. Also, in this study unlike generic earthquake awareness tools [34,35], the EKQ: Targets three specific domains (Geological knowledge, Mitigation measures, and preparedness knowledge) with evidence-based items, emphasizes preventable knowledge (e.g., retrofitting, secondary hazards) rather than basic facts and integrates cultural relevance. As compared to previous tools, which barely integrate in-depth information and actual action [36], the EKQ developed herein is aligned with the UNDRR disaster management guidelines. It aims to identify public awareness deficits and guide targeted education campaigns in facilitating heightened disaster preparedness, response, and recovery [37].

Earthquake risks and mitigation strategies vary across regions, making it challenging to develop a tool that is universally applicable. Additional validation studies in different cultural and geographic contexts are recommended. In Iran, a country with numerous active fault lines, understanding this factor is critical for public awareness and preparedness. The inclusion of culturally relevant examples of local fault lines could further enhance the questionnaire’s applicability.

The developing and validation of the EKQ is a great step towards enhancing public earthquake awareness and disaster preparedness in line with the UNDRR recommendations. By creating a psychometrically valid instrument through a rigorous process of systematic reviews, expert interviews, and exploratory and confirmatory factor analyses, the current study provides a reliable and valid instrument for assessing earthquake knowledge in three significant areas of geological knowledge, mitigation strategies, and behavioral preparedness. Collectively, these areas explained 83.6% of the variance in earthquake awareness, reflecting the EKQ’s adequacy for accounting for significant aspects of public knowledge. The study’s identification of precise knowledge gaps, particularly in the areas of structural safety and safe location awareness, offers practical feedback for the development of targeted education campaigns. Placing the EKQ within Tabriz, Iran’s seismic and cultural context while ensuring methodological rigor validity to the UNDRR’s imperative for evidence-based disaster risk reduction strategies. The EKQ may inform policymakers and educators in the development of interventions to enhance earthquake preparedness, response, and recovery, and ultimately decrease earthquake vulnerability in seismically active areas.

In summary, compared to existing tools such as the Earthquake Knowledge Assessment Scale (EKAS) [19]and other disaster awareness measures [34,35], the present EKQ demonstrates three key advancements. First, in terms of construct coverage, the EKQ incorporates a broader range of domains geological knowledge, mitigation measures, and preparedness knowledge, while many existing tools primarily focus on general awareness or basic safety tips. Second, regarding psychometric robustness, the EKQ underwent a rigorous validation process using polychoric EFA, parallel analysis, and CFA, supported by multiple reliability indices (Cronbach’s Alpha, McDonald’s Omega, ICC), whereas many prior instruments lack such a comprehensive psychometric evaluation [21,34]. Finally, in terms of cultural tailoring, the EKQ was developed through expert interviews and face/content validation among Persian-speaking populations, ensuring linguistic clarity and contextual relevance an area often overlooked in generic or translated tools. These contributions position the EKQ as a culturally grounded and psychometrically sound instrument for assessing actionable earthquake knowledge in high-risk populations.

## Study strengths and limitations

Among the strengths of our study is the strict factor analysis procedure utilized in validating the three-factor solution of earthquake preparedness. Kaiser’s criterion (eigenvalues ≥ 1.0) initially suggested a three-factor solution, and this was reinforced by parallel analysis with 1,000 random data sets and Velicer’s Minimum Average Partial (MAP) test, which added reliability and avoided drawbacks of Kaiser’s rule. Items were selected on a criterion basis of loading patterns and theoretical significance, and items with cross-loadings or loadings <0.2 were dropped for factor clarity. Items 6 (0.284) and 9 (0.282), while having lower loadings, were exceptionally left in since they had acceptable item-total correlations (Item 6: r = 0.41; Item 9: r = 0.38) and invariant model fit indices (CFI = 0.92, RMSEA = 0.06) when tested for deletion. Item 6, awareness of closeness to geological knowledge, theoretically strengthens Factor 1 by accessing a key aspect of risk perception highly relevant in Iran’s earthquake-prone context. Similarly, Item 9, measuring media consumption, enriches Factor 3 (preparedness knowledge) by incorporating active safety awareness, strengthening its conceptual scope. The article presents internal consistency testing through the utilization of both McDonald’s Omega and Cronbach’s Alpha, with the findings of both of these measures reflecting acceptable reliability. The employment of both is explained as Cronbach’s Alpha has widespread acceptance and enables comparison of results with other research, whereas McDonald’s Omega provides a more rigorous test for factor models, and hence aligns with the research approach utilized. Utilizing both measures provides improved interpretability and validity of results within varying statistical paradigms [38].

A limitation of the study is the relatively low factor loadings of Items 6 (0.284) and 9 (0.282), which, while statistically and theoretically justifiable, could reflect weaker relationships to their respective factors than more highly loading items. This could reduce the precision of these factors in assessing the intended constructs. Item refinement for these items or the inclusion of additional measures could be considered in future studies to improve factor loadings without sacrificing theoretical relevance. Additionally, this research, restricted to adults 18+ in Tabriz, Iran, might not apply to other areas or age groups, and its EKQ might need modification for other cultures or seismological environments. Utilizing a three-point Likert scale might also restrict the sensitivity of measuring knowledge levels in detail. Another limitation is the potential sampling bias due to restricting the study population to a single city, Tabriz. Although the city is highly relevant due to its seismic risk, relying on one geographic and cultural context may limit the generalizability of the findings. Future validation efforts should therefore include samples from other regions of Iran with varying socio-demographic characteristics to enhance the external validity of the EKQ. The other limitation of the present study is the relatively small sample size (n = 20) used for test-retest reliability analysis. Although sufficient for preliminary assessment, future studies should replicate this analysis with a larger and more diverse sample to ensure the stability of the instrument across broader populations.

Additionally, due to the self-reported format of the EKQ, participants may have provided responses influenced by social desirability bias, especially in a culturally sensitive domain like disaster preparedness, where appearing knowledgeable may be perceived as socially or morally favorable. Future studies are encouraged to incorporate methods for behavioral assessments to address this limitation.

## Conclusion

The testing of the psychometric properties of the EKQ, developed for the Persian culture, indicates strong validity and reliability in measuring earthquake knowledge. The EFA yielded three significant factors: Geological knowledge, Mitigation measures, and preparedness knowledge, explaining 83.6% of the variance, with factor 1 (Geological knowledge) as the largest contributor (58.52%). The greater factor loadings, particularly on Geological knowledge (0.958 on the fault line connection item), and reliability estimates indicate the internal consistency of the questionnaire. The CFA also established the model fit, with satisfactory fit indices, although the chi-square was significant and sample size impacted. The questionnaire is a valid and reliable tool for measuring earthquake awareness among Persian populations and could be utilized for informing earthquake education and preparedness campaigns. The study’s EKQ makes a significant contribution to earthquake education by providing a validated tool to assess public awareness across geological knowledge, mitigation strategies, and preparedness knowledge, aligning with UNDRR guidelines. The EKQ can become part of school curricula or community workshops in earthquake-risk areas like Tabriz to promote targeted, evidence-based education to strengthen preparedness, response, and recovery. Given its validated structure and contextual relevance, the EKQ can be integrated into national disaster preparedness strategies, public education campaigns, and school curricula across earthquake-prone regions in Iran to enhance public awareness, promote preventive behaviors, and support evidence-based policymaking.
